# Identification of prognostic biomarkers in papillary renal cell carcinoma and PTTG1 may serve as a biomarker for predicting immunotherapy response

**DOI:** 10.1080/07853890.2021.2011956

**Published:** 2022-01-17

**Authors:** Xi Tian, Wen-Hao Xu, Fu-Jiang Xu, Hui Li, Aihetaimujiang Anwaier, Hong-Kai Wang, Fang-Ning Wan, Yu- Zhu, Da-Long Cao, Yi-Ping Zhu, Guo-Hai Shi, Yuan-Yuan Qu, Hai-Liang Zhang, Ding-Wei Ye

**Affiliations:** a Department of Urology, Fudan University Shanghai Cancer Center, School of Life Sciences, Fudan University, Shanghai, P.R. China; b Department of Oncology, Shanghai Medical College, Fudan University, Shanghai, P.R. China; c Department of Endocrinology, Changhai Hospital, Naval Medical University, Shanghai, P.R. China

**Keywords:** Papillary renal cell carcinoma, PTTG1, biomarker, prognosis, immune checkpoint blockade

## Abstract

**Objective:**

This study aims to identify potential prognostic and therapeutic biomarkers in papillary renal cell carcinoma (pRCC).

**Methods:**

Two microarray datasets were downloaded from the Gene Expression Omnibus (GEO) database and differentially expressed genes (DEGs) were identified. The protein-protein interaction (PPI) networks and functional annotations of DEGs were established. Survival analysis was utilized to evaluate the prognostic significance of the DEGs and the association between the expression level of candidate biomarkers and various tumour-infiltrating immune cells was explored. The role of *PTTG1* in tumour microenvironments (TME) was further explored using Single-cell RNA-seq and its prognostic and therapeutic significance was validated in Fudan University Shanghai Cancer Centre (FUSCC) cohort.

**Results:**

Eight genes, including *BUB1B*, *CCNB1*, *CCNB2*, *MAD2L1*, *TTK*, *CDC20*, *PTTG1,* and *MCM* were found to be negatively associated with patients’ prognosis. The expression level of *PTTG1* was found to be significantly associated with lymphocytes, immunomodulators, and chemokine in the TCGA cohort. Single-cell RNA-seq information indicated that *PTTG1* was strongly associated with the proliferation of T cells. In the FUSCC cohort, the expression level of PTTG1 was also statistically significant for both progression-free survival (PFS) and overall survival (OS) prediction (HR = 2.683, *p* < .001; HR = 2.673, *p* = .001). And higher expression level of PTTG1 was significantly associated with immune checkpoint blockade (ICB) response in the FUSCC cohort (*χ*^2^=3.99, *p*** **<** **.05).

**Conclusions:**

Eight genes were identified as a prognostic biomarker and the expression level of PTTG1 was also found to serve as a potential predictor for ICB response in pRCC patients.

**Key messages:**
- Eight genes, including *BUB1B*, *CCNB1*, *CCNB2*, *MAD2L1*, *TTK*, *CDC20*, *PTTG1,* and *MCM* were found to be negatively associated with pRCC patients’ prognosis.
- Expression level of PTTG1 was significantly associated with tumour microenvironment including lymphocytes, immunomodulators, and chemokines.
- Higher expression level of PTTG1 was significantly associated with immune checkpoint blockade (ICB) response in FUSCC cohort

## Introduction

Papillary renal cell carcinoma (pRCC), the second most common histologic subtype of renal cell carcinoma, originates from tubular epithelial cells, accounts for about 10%-15% of all renal tumours [1,2]. The histological features of pRCC are fibrovascular cores with a papillary arrangement of tumour cells [2]. pRCC can be divided into two subtypes: type I and type II. Type 1 pRCC was characterized by monolayer, light staining, and basophilic small cells, often with rich foam macrophage infiltration, and type II showed high Fuhrman grade, eosinophilic cytoplasm, and pseudostratified arrangement in the centre of the nipple [3].

Recent studies have shown that type II pRCC is a heterogeneous tumour, which can be further subdivided into other subtypes according to the genetic and molecular composition of the tumour, reflecting different clinical courses and prognoses [4]. It is reported that pRCC type II patients have a worse outcome than clear cell renal cell carcinoma (ccRCC) [5,6] and it is difficult to predict the prognosis of patients. Some genetic features of pRCC have been recognized such as mutations of MET, NF2, SETD2, and Nrf2 pathway genes [7–9]. However, these mutations were found in only 10% to 15% of pRCC tumours in these studies [10,11]. In recent years, with a better understanding of RCC molecular biology, targeted drugs, and therapeutic effects have been improved [12].

With the rapid development of microarray technology and bioinformatics analysis, our understanding of differentially expressed genes (DEGs) and functional pathways related to the occurrence and development of pRCC has become more comprehensive. However, the rarity of this tumour has become an obstacle in identifying potential markers to distinguish pRCC and provide potential therapeutic targets. In this study, two mRNA microarray data sets were downloaded from Gene Expression Omnibus (GEO) [13] for analysis to obtain DEGs between cancer tissues and normal adjacent tissues. Subsequently, to further understand the molecular mechanism of tumorigenesis, gene ontology (GO) [14] and Kyoto genome encyclopaedia (KEGG) [15] pathway enrichment analyses were carried out. The protein-protein interaction (PPI) network reveals the specific functions of all proteins and describes the importance of these interactions in biological processes, molecular functions, and signal transduction. As the introduction of immune checkpoint blockade (ICB) into the treatment of RCC has transformed the therapeutic landscape in this recalcitrant disease, tumour microenvironments (TME) variation of pRCC may influence ICB response. Thus, we explored the association between the candidate genes and tumour infiltrating immune cells and investigate the potential ability of PTTG1 expression in predicting ICB response in pRCC patients.

To determine the candidate biomarkers and their possible role in pRCC, this work focussed on analyzing gene expression profiles, assessing the prognostic significance, and exploring potential biological alterations. Furthermore, the associations between TME and the hub genes were explored and real-world data were obtained to verify the potential therapeutic values of *PTTG1*. Our findings may shed light on the clinical management of pRCC.

## Materials and methods

### Original biological microarray data

Gene Expression Omnibus (GEO) is a public functional genomic database that stores high throughput gene expression data, chips, and microarrays. The original gene expression microarray data (GSE48352 and GSE26574) were obtained from GEO, for patients with pRCC. Transcriptional and corresponding clinical information of 323 pRCC patients were also obtained from The Cancer Genome Atlas (TCGA, https://portal.gdc.cancer.gov/).

### Screening of DEGs and construction of PPI networks

The differentially expressed genes (DEGs) between tumour and adjacent normal tissues were identified by GEO2R. DEGs with log|FC| (fold change) ≥1 and *p*-value <.01 were considered statistically significant. Search Tool for the Retrieval of Interacting Genes (STRING; http://string-db.org) (version 10.0) online database was utilized to predict protein-protein interaction (PPI) networks of DEGs [16]. This may help to further understand the underlying mechanisms of the development and progression of pRCC. Cytoscape (version 3.5) [17] and Cytoscape’s plug-in Molecular Complex Detection (MCODE) (version 1.4.2) were used to explore the most important DEGs [18]. The most important modules in the PPI network are selected as follows: MCODE Score >24.

### Hub genes selection and functional enrichment analysis

The eight genes with the highest MCODE Score in the PPI network were defined as hub genes. Biological properties such as biological processes (BP), molecular functions (MF), and cellular components (CC) were extracted from gene ontology (GO) enrichment analysis to determine the role of DEGs in pRCC. Kyoto Encyclopaedia of Genes and Genomes (KEGG) is a database resource for understanding high-level functions and biological systems from large-scale molecular datasets generated by high-throughput experimental technologies. Functional enrichment analysis of the eight hub genes was performed using the ClusterProfiler package [19] and *p*-value < .05 is considered statistically significant.

### Analyzing prognostic values of hub genes and exploring potential immune-related alterations in TCGA cohort

Kaplan-Meier method (Cutoff values were taken using X-tile software [20]) and univariate Cox regression analysis were utilized to explore the potential prognostic value of the hub genes. Multivariate cox regression analysis was also performed with Cox logistic regression models including age (ref. <60 years), gender (ref. Male), laterality (ref. Right), pT stage (ref. T1-T2), pN stage (ref. N0), pM stage (ref. M0) and gene expression (ref. Low). C-indexes were calculated to assess the prognostic models. The association between hub genes and various immune cells was explored by using TIMER [21] and *p* < .05 was considered significant.

### Exploring the role of PTTG1 in TME and gene set enrichment analysis

As the expression level of *PTTG1* was significantly associated with almost all the explored immune cells abundance, further analyses were performed to explore associations between *PTTG1* and TME. TISIDB [22] was utilized to explore the potential associations between *PTTG1* expression and TME including lymphocytes, immunomodulators, and chemokines. Single-cell RNA-seq (scRNA-seq) enables a better understanding of TME and TISCH [23] was utilized to explore the role of *PTTG1* in TME. Due to the lack of scRNA-seq of pRCC, three datasets containing scRNA-seq of ccRCC (GSE145281, GSE139555, and GSE111360) were enrolled for further analysis. Gens set enrichment analysis (GSEA) was also used to explore the potential biological changes caused by *PTTG1*.

### Exploring the prognostic and therapeutic significance of PTTG1 in FUSCC cohort

This study included 126 pRCC patients (Table 1) who underwent surgical treatment from Fudan University Shanghai Cancer Centre (FUSCC) between 2007 and 2020 and tumour specimens were obtained with informed consent. Rabbit anti-PTTG1 monoclonal antibody was utilized (ab128040, Abcam, USA) to detect the expression level of PTTG1 by using immunohistochemistry (IHC). Positive or negative staining of a certain protein in an FFPE slide was independently evaluated by two experienced pathologists and determined as follows. The overall IHC score from 0 to 12 was evaluated according to the multiply of the staining intensity and extent score [24]. According to the IHC score, the patients were divided into two groups: high expression group (IHC score > 3) and low expression group (IHC score ≤ 3) of *PTTG1*. Correlation analyses between the expression level of PTTG1 and clinicopathological features were carried out by chi-square test. In the FUSCC cohort, 62 pRCC patients previously treated with ICB treatment were enrolled for further analysis to explore the potential reference value of the PTTG1 expression level.

**Table 1.** Clinicopathological characteristics at surgery in relation to *PTTG1* expression status in pRCC patients from FUSCC cohort.

Variable	Entire group (*n* = 126)	*PTTG1* expression		*χ* ^2^	*p-*value
		Low expression (*n* = 63)	High expression (*n* = 63)		
Age at surgery (y, median ± SD)		57.0 ± 11.7	57.0 ± 13.2		
BMI (kg/m^2^, median ± SD)		22.9 ± 3.4	22.5 ± 3.2		
Tumour size (cm, median ± SD)		4.0 ± 2.5	5.0 ± 3.0		
OS time (month, median ± SD)		54.0 ± 35.6	42.0 ± 35.2		
PFS time (month, median ± SD)		54.0 ± 38.7	36.0 ± 39.7		
Sex (*n*, %)				0.904	.342
Male	85, 67.5%	45, 71.4%	40, 63.5%		
Female	41, 32.5%	18, 28.6%	23, 36.5%		
Laterality (*n*, %)				1.581	.209
Left	71, 56.3%	39, 61.9%	32, 50.8%		
Right	55, 43.7%	24, 38.1%	31, 49.2%		
Tumour type				0.293	.588
Type 1 pRCC	53, 42.1%	28, 44.4%	25, 39.7%		
Type 2 pRCC	73, 57.9%	35, 55.6%	38, 60.3%		
T stage (*n*, %)				3.889	**.049**
T1–T2	90, 71.4%	50, 79.4%	40, 63.5%		
T3–T4	36, 28.6%	13, 20.6%	23, 36.5%		
N stage (*n*, %)				6.038	**.014**
N0	101, 80.2%	56, 88.9%	45, 71.4%		
N1	25, 19.8%	7, 11.1%	18, 28.6%		
M stage (*n*, %)				5.704	**.017**
M0	99, 78.6%	55, 87.3%	44, 69.8%		
M1	27, 21.4%	8, 12.7%	19, 30.2%		
AJCC stage (*n*, %)				7.327	**.007**
I–II	73, 57.9%	44, 69.8%	29, 46.0%		
III–IV	53, 42.1%	19, 30.2%	34, 54.0%		
Furhman grade (*n*, %)				7.966	**.005**
1–2	27, 21.4%	20 , 31.7%	7, 11.1%		
3–4	99, 78.6%	43, 68.3%	56, 88.9%		
ICB response (*n* = 62)		*n* = 19	*n* = 43	3.99	**.046**
PD/SD	41, 66.1%	16, 84.2%	25, 58.1%		
PR/CR	21, 33.9%	3, 15.8%	18, 41.9%		

pRCC: papillary renal cell carcinoma; FUSCC: Fudan University Shanghai Cancer Centre; BMI: body mass index; AJCC: American Joint Committee on Cancer; ICB: immune checkpoint blockade. *p-value less than .05 was considered as statistically significant and marked in bold.

## Results

### Identification of DEGs and PPI networks construction

**Figure 1.**
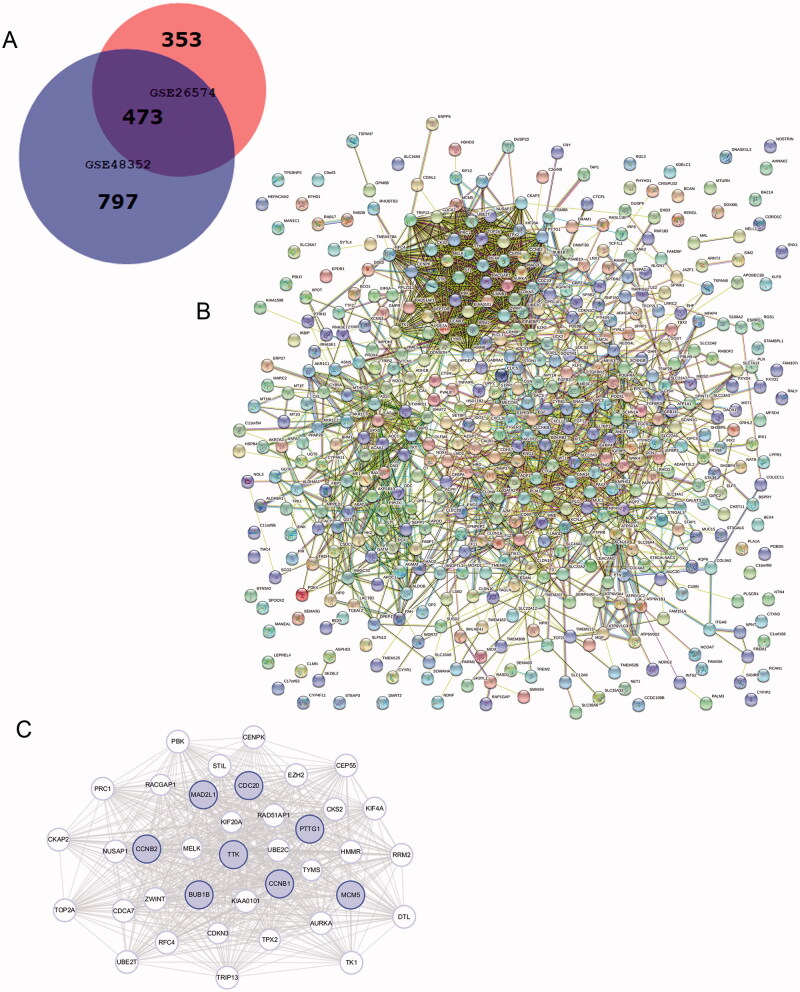
Venn diagram, PPI network, and the most significant module of DEGs. (A) DEGs were selected with a fold change >2 and *p*-value <.01 among the mRNA expression profiling chip datasets GSE48352 and GSE26574. The 2 datasets show an overlap of 473 genes in the Venn diagram. (B) The PPI network of DEGs was constructed using STRING. (C) The most significant module was obtained from the PPI network with 38 nodes. Significant edges are related to the cell cycle and they are marked in light blue.

1270 DEGs were identified in GSE48352, while 826 DEGs were founded in GSE26574. As shown in the Venn diagram, there are common 473 DEGs between tumour tissues and adjacent normal tissues (Figure 1(A)). PPI network of DEGs was built (Figure 1(B)) and the most important modules were identified using the Cytoscape plugin (Figure 1(C)). 38 functionally related hub genes of network include *PTTG1*, *CDC20*, *TPX2*, *AURKA*, *MAD2L1*, *TRIP13*, *CCNB2*, *KIF20A*, *CKS2*, *CCNB1*, *UBE2C*, *BUB1B*, *ZWINT*, *RACGAP1*, *TTK*, *HMMR*, *TOP2A*, *TYMS*, *TK1*, *KIF4A*, *PRC1*, *PBK*, *NUSAP1*, *EZH2*, *RFC4*, *DTL*, *CENPK*, *MCM5*, *MELK*, *UBE2T*, *RRM2*, *CEP55*, *KIAA0101*, *RAD51AP1*, *CDCA7*, *CKAP2*, *CDKN3* and *STIL*. And the eight hub genes are as follows: *BUB1B*, *CCNB1*, *CCNB2*, *MAD2L1*, *TTK*,*CDC20*, *PTTG1*, *MCM5*.

### Validating DEGs in TCGA cohort and functional enrichment analysis

**Figure 2.**
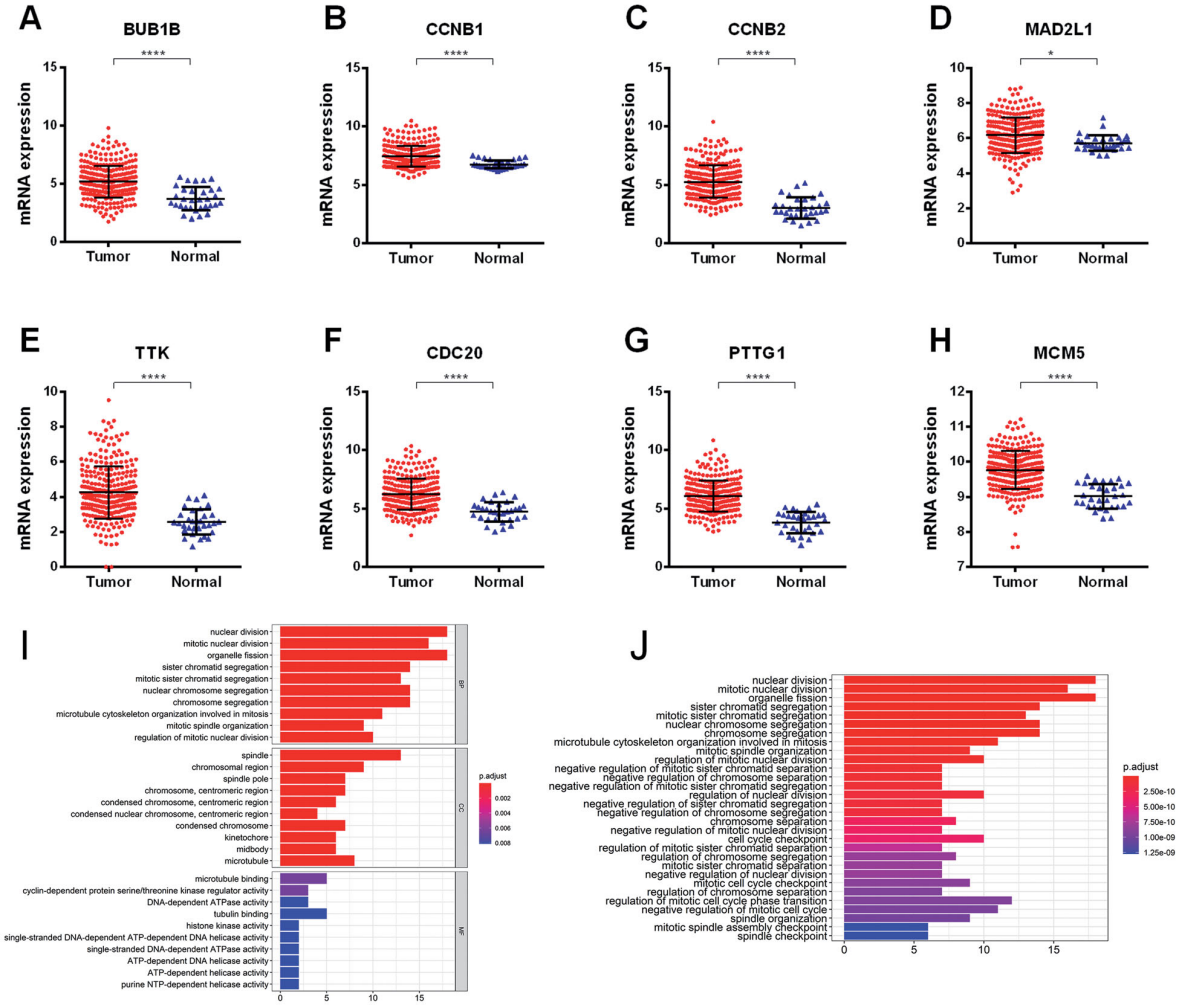
Validating DEGs in TCGA cohort. The expression levels of the eight hub genes were relatively higher in tumour tissues compared to normal tissues (A–H). Functional enrichment analysis results indicated that the 38 DEGs were functionally enriched in the nuclear division, spindle and microtubule-binding, etc. (I–J).

The expression levels of the eight hub genes were relatively higher in tumour tissues compared to normal tissues (Figure 2(A–H)). Functional enrichment analysis results indicated that the 38 DEGs were functionally enriched in the nuclear division, spindle, and microtubule-binding, etc. (Figure 2(I,J)). The main functional enrichment results of 38 DEGs are listed in Table 2.

**Table 2.** GO and KEGG pathways enrichment analysis of DEGs in the most significant module.

Term	Description	Gene ratio	*p-*value (adjusted)
GO:0000280	Nuclear division	18/37	6.37E-18
GO:0140014	Mitotic nuclear division	16/37	6.37E-18
GO:0048285	Organelle fission	18/37	1.59E-17
GO:0005819	Spindle	13/37	2.51E-12
GO:0098687	Chromosomal region	9/37	5.13E-07
GO:0000922	Spindle pole	7/37	5.59E-07
GO:0008017	Microtubule-binding	5/35	.006378744
GO:0016538	Serine/threonine kinase regulator activity	3/35	.006378744
GO:0008094	DNA-dependent ATPase activity	3/35	.008105314

GO: Gene Ontology; KEGG: Kyoto Encyclopaedia of genes and genomes; DEGs: differentially expressed genes.

### The eight hub genes were of prognostic values

**Figure 3.**
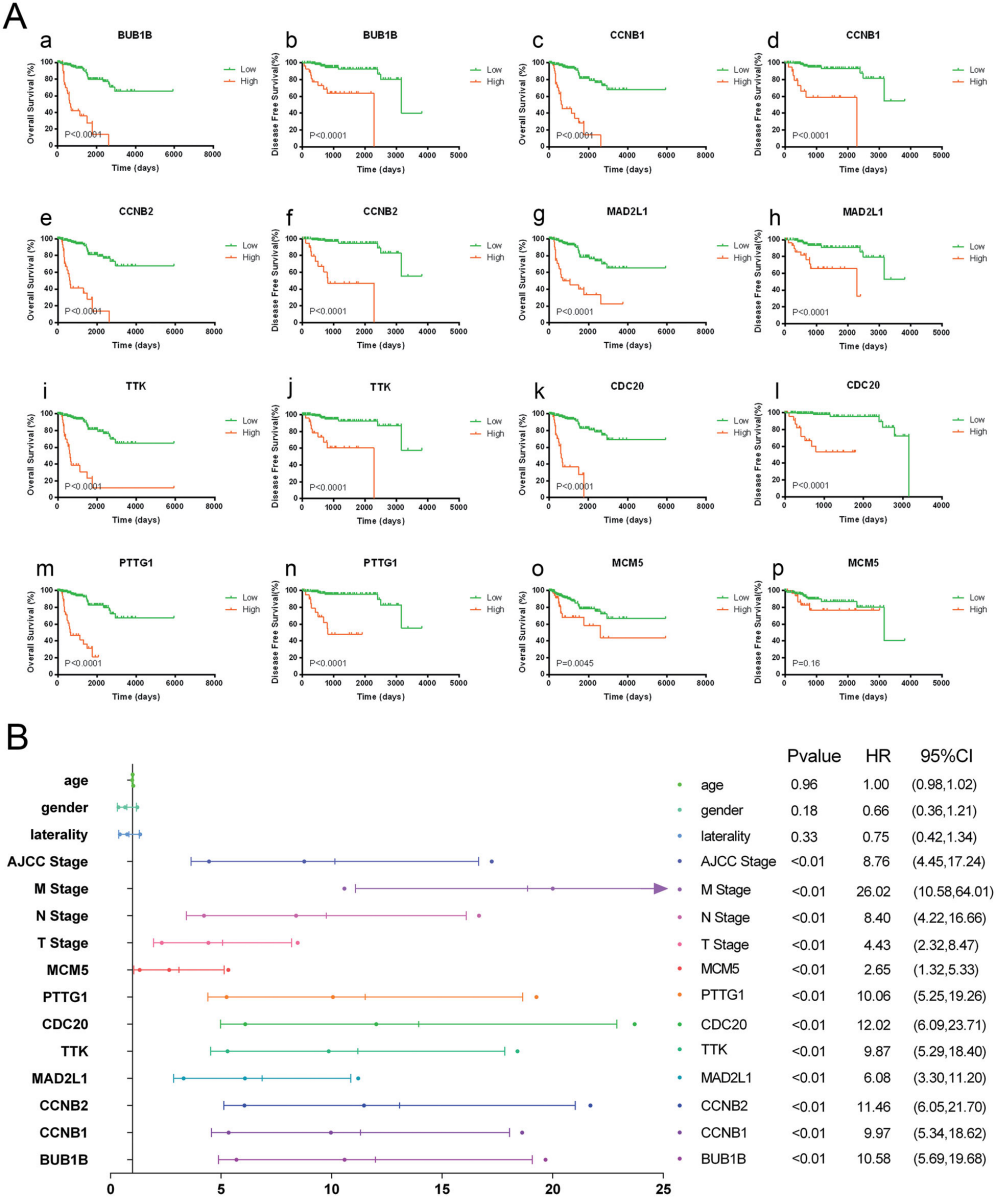
Eight hub genes were of prognostic values. The overall survival (OS) of pRCC patients with elevated expression of the eight hub genes was significantly worse. And apart from MCM5, the higher expression level of the other seven genes including BUB1B, CCNB1, MAD2L1, TTK, CDC20, MCM5, PTTG1 was significantly associated with progression-free survival (A). Univariate regression analyses indicated that the expression level of the eight hub genes, AJCC stage, pTNM stage were significantly associated with OS (*p* < .05) (B).

**Figure 4.**
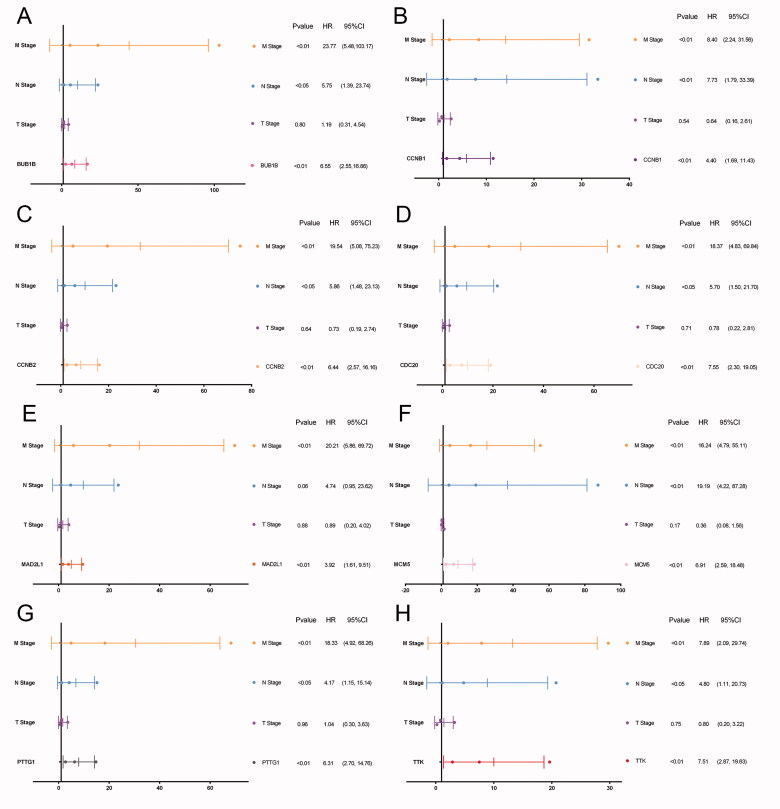
Multivariate cox regression analyses of eight hub genes. Multivariate regression analyses indicated that expression levels of the eight hub genes were still significantly associated with OS (*p* < .05; A–H).

In TCGA cohort, 51 patients were recorded as died and the overall survival (OS) of pRCC patients with elevated expression of the eight hub genes was significantly worse. And apart from MCM5, the higher expression level of the other seven genes including *BUB1B*, *CCNB1*, *MAD2L1*, *TTK*, *MCM5*, *CDC20*, *PTTG1* was significantly associated with progression-free survival (PFS) (Figure 3(A)). Univariate cox regression analyses indicated that the expression level of the eight hub genes, AJCC stage, pTNM stage were significantly associated with OS (*p* < .05; Figure 3(B)). Multivariate cox regression analyses indicated that expression levels of the eight hub genes were still significantly associated with OS (*p* < .05; Figure 4(A–H)). We have calculated the c-indices (Table 3) of eight prognostic models containing the TNM stage and expression level of each hub genes. The c-indices were all increased when adding the expression level of hug genes and it range from 0.825 to 0.8971, which indicated the stability of the eight biomarkers.

**Table 3.** C-indexes of multivariate cox models in TCGA cohort.

Variables in multivariate cox model	C-index	Standard deviation
T stage, N stage, M stage	0.7956	0.0512
T stage, N stage, M stage, expression level of BUB1B	0.8971	0.0304
T stage, N stage, M stage, expression level of CCNB1	0.8575	0.0422
T stage, N stage, M stage, expression level of CCNB2	0.8615	0.0426
T stage, N stage, M stage, expression level of MAD2L1	0.825	0.0554
T stage, N stage, M stage, expression level of TTK	0.8637	0.0427
T stage, N stage, M stage, expression level of CDC20	0.8663	0.0422
T stage, N stage, M stage, expression level of PTTG1	0.8826	0.0323
T stage, N stage, M stage, expression level of MCM5	0.8491	0.0433

### PTTG1 may play a key role in the tumour microenvironment

**Figure 5.**
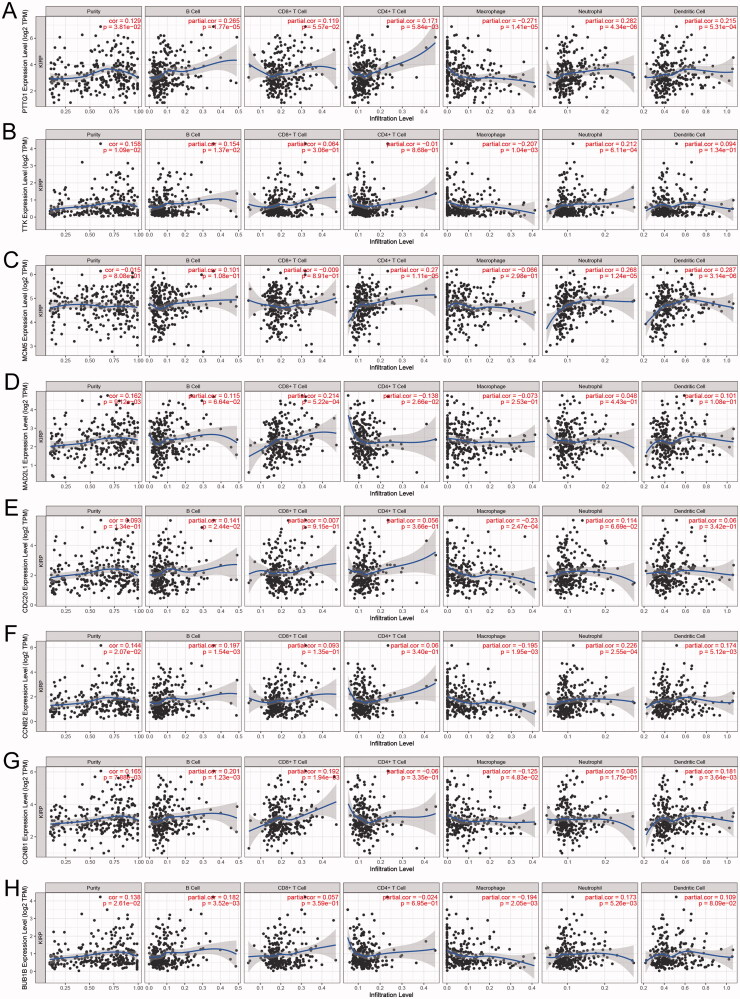
Associations between PTTG1 and immune cells. Within the eight hub genes, expression levels of some genes are significantly associated with tumour purity and various immune cells abundance including B cells, CD8+ T cells, CD4+ T cells, macrophages, neutrophils, and dendritic cells (A–H). Notably, the expression level of PTTG1 was significantly associated with almost all the explored immune cells abundance except for CD8+ T cells (A).

**Figure 6.**
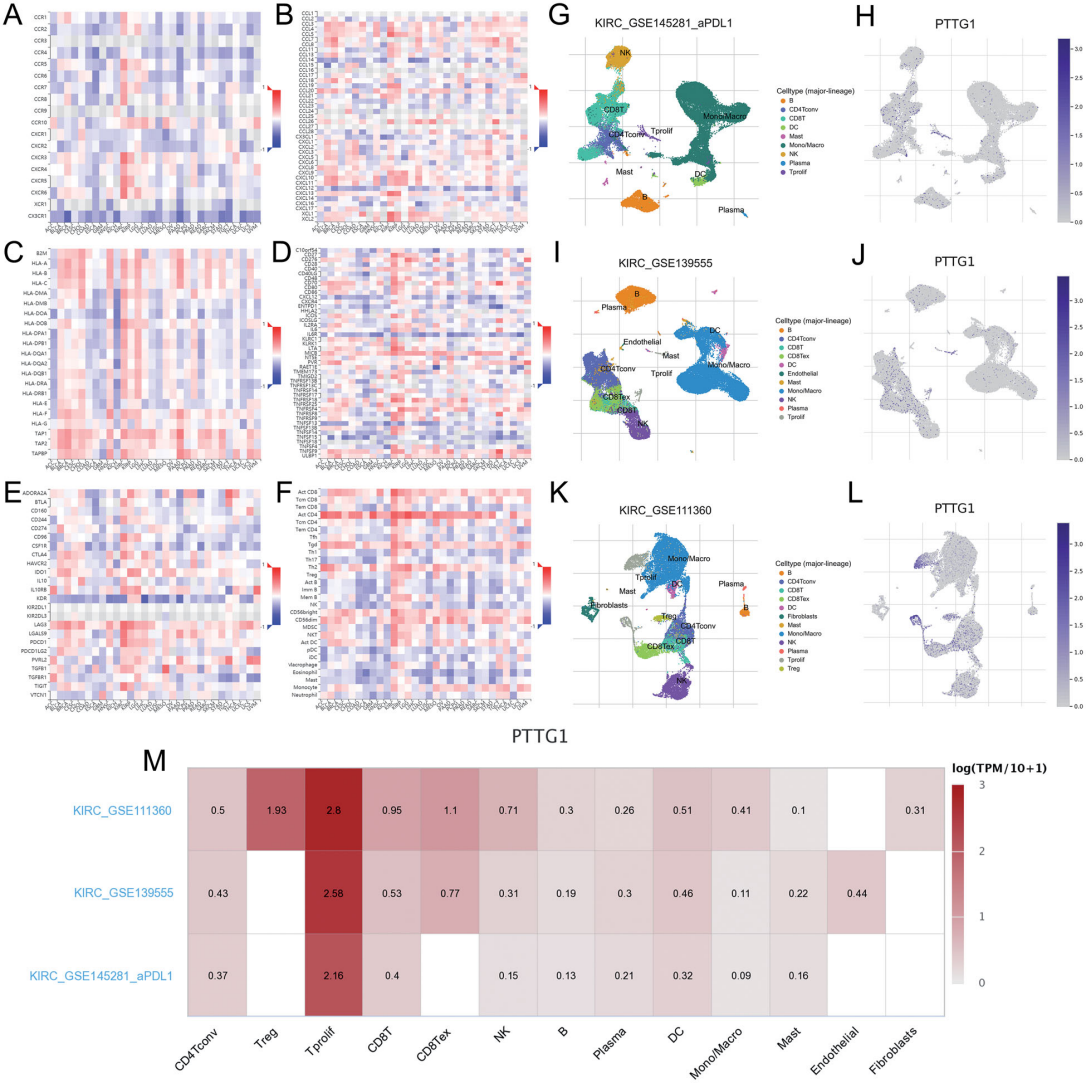
Further exploration of PTTG1 and TME. (A–F) PTTG1 was found to be positively associated with chemokine receptors (CXCR3, CXCR5, etc.), chemokines (CCL5, CXCL13, etc.), major histocompatibility complex molecules (TAPBP, HLA-DOB, etc.), immune stimulators (TNFRSF4, LTA, etc.), immune inhibitors (LAG3, PDCD1, IDO1, etc.) and lymphocytes (activated CD8+ T cells, activated CD4+ T cells, etc.). Analyses of scRNA-seq indicated (G–L) that PTTG1 was significantly associated with the proliferation of T cells in all three datasets (GSE145281, GSE139555, and GSE111360). In addition, PTTG1 may be also associated with regulatory T cells and exhausted CD8 T Cells (M).

**Figure 7.**
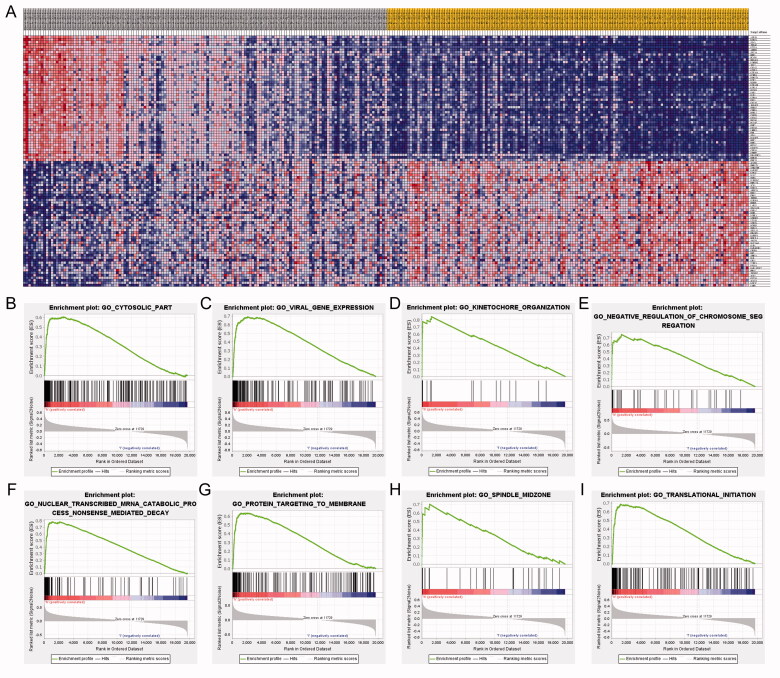
GESA results. GSEA indicated that PTTG1 may influence the expression pattern of pRCC (A) and a higher expression level of *PTTG1* was significantly associated with viral gene expression, nuclear-transcribed mRNA catabolic process, negative regulation of chromosome segregation, cytosolic part, etc. (B–I).

Within the eight hub genes, expression levels of some genes are significantly associated with tumour purity and various immune cells abundance including B cells, CD8^+^ T cells, CD4^+^ T cells, macrophages, neutrophils, and dendritic cells (Figure 5(A–H)). Notably, the expression level of *PTTG1* was significantly associated with almost all the explored immune cells abundance except for CD8^+^ T cells (Figure 5(A)). Thus, further analyses were carried out to explore the associations between *PTTG1* and TME. As shown in Figure 6(A–F), *PTTG1* was found to be positively associated with chemokine receptors (*CXCR3*, *CXCR5*, etc), chemokines (*CCL5*, *CXCL13*, etc), major histocompatibility complex molecules (*TAPBP*, *HLA-DOB*, etc), immune stimulators (*TNFRSF4*, *LTA*, etc), immune inhibitors (*LAG3*, *PDCD1*, *IDO1*, etc) and lymphocytes (activated CD8^+^ T cells, activated CD4^+^ T cells, etc). Analyses of scRNA-seq indicated (Figure 6(G–L)) that *PTTG1* was significantly associated with the proliferation of T cells in all three datasets (GSE145281, GSE139555, and GSE111360). Thus, a higher expression level of *PTTG1* may indicate a higher infiltration of T cells. Since T cells play a key role in antitumor immunity, *PTTG1* may be a biomarker for predicting ICB response. In addition, *PTTG1* may be also associated with regulatory T cells and exhausted CD8 T Cells (Figure 6(M)). GSEA indicated that PTTG1 may influence the expression pattern of pRCC (Figure 7(A)) and a higher expression level of *PTTG1* was significantly associated with viral gene expression, nuclear-transcribed mRNA catabolic process, negative regulation of chromosome segregation, cytosolic part, etc. (Figure 7(B–I)). The detailed results of GSEA were listed in Table S1.

### Validation and exploration of the potential prognostic and therapeutic significance of PTTG1

**Figure 8.**
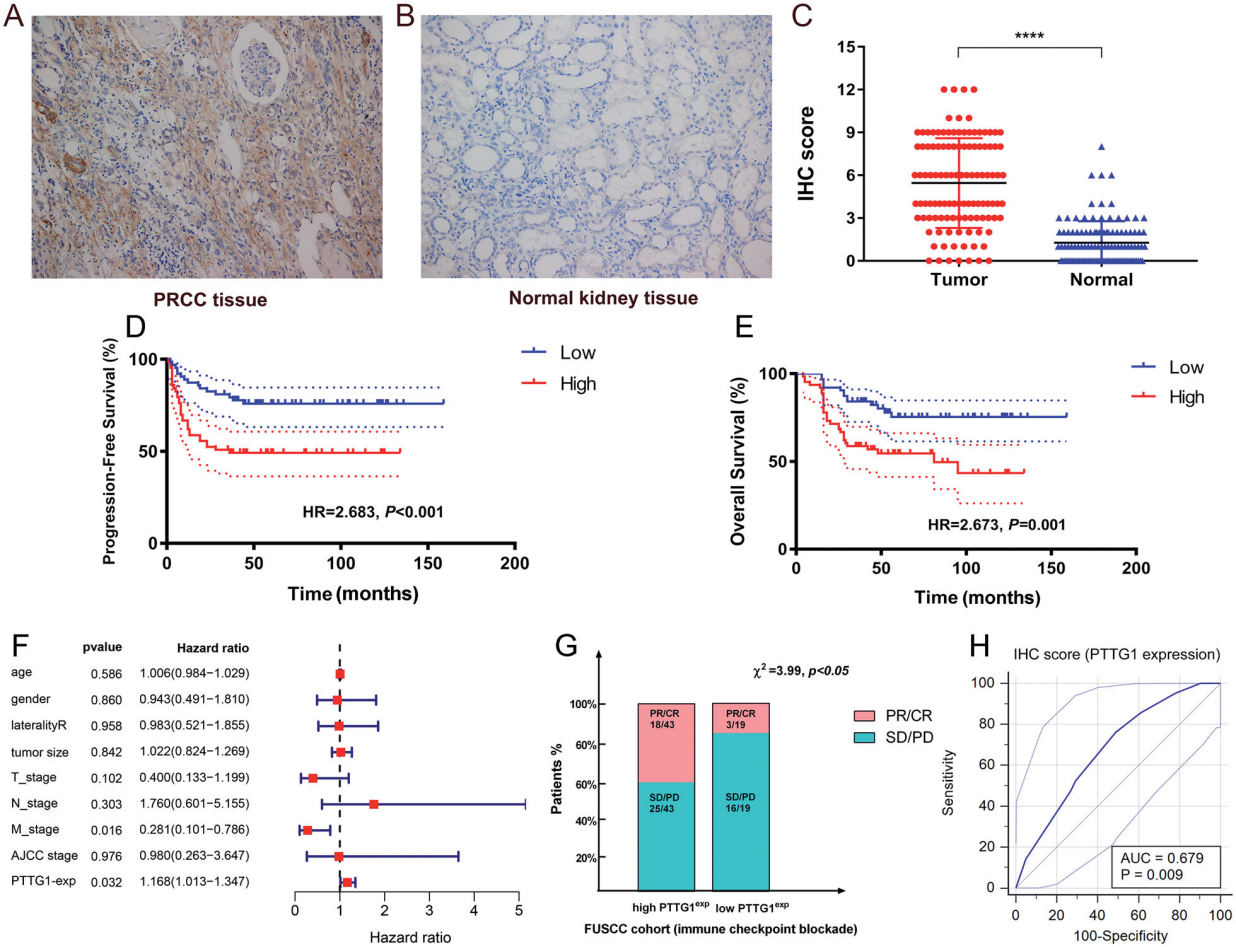
Validation and exploration of the potential prognostic and therapeutic significance of PTTG1 in FUSCC cohort. The expression level of PTTG1 was significantly higher in pRCC tissues compared with adjacent normal tissues in FUSCC cohort (A–C). Survival analyses (D–E) show that elevated expression level of PTTG1 was significantly associated with poorer OS (HR = 2.673, *p* = .001) and PFS (HR = 2.683, *p* < .001). The retrospective analysis (F–G) indicated that elevated PTTG1 expression was significantly associated with better ICB response (*χ*^2^=3.99, *p* < .05) and PTTG1 may serve as a stable biomarker for immunotherapy (AUC = 0.679, *p* = .009).

To validate the potential prognostic and therapeutic significance of *PTTG1*, we explored the relative expression level of PTTG1 using IHC in the FUSCC cohort (Table 4). Results indicated that the expression level of PTTG1 was significantly higher in pRCC tissues compared with adjacent normal tissues in the FUSCC cohort (Figure 8(A–C)). 46 patients were recorded as died in the FUSCC cohort and survival analyses (Figure 8(D–E)) show that elevated expression level of PTTG1 was significantly associated with poorer OS (HR = 2.673, *p* = .001) and PFS (HR = 2.683, *p* < .001). In addition, increased *PTTG1* expression in pRCC patients significantly associated with advanced pT (*p* = .049), pN (*p* = .014), and pM stage (*p* = .017), AJCC stage (*p* = .007) and Furhman grade (*p* = .005) in the FUSCC cohort (Table 1). Univariate Cox regression analysis of FUSCC cohorts was listed in Table 5. As depicted in Figure 8(F), PTTG1 expression was significantly associated with overall survival in multivariate regression in the FUSCC cohort. Thus, the independent prognostic significance of PTTG1 was validated. Thank you again for your professional suggestions. The retrospective analysis (Figure 8(G,H)) indicated that elevated PTTG1 expression was significantly associated with better ICB response (*χ*^2^=3.99, *p* < .05) and PTTG1 may serve as a stable biomarker for immunotherapy (AUC = 0.679, *p* = .009). The c-index of the prognostic model was increased when adding the expression level of PTTG1 in the FUSCC cohort (Table 6), which also indicated the stable predicting ability of PTTG1. As a higher PTTG1 expression level was significantly associated with ICB response, high PTTG1 expression may be also associated with a better prognosis among pRCC patients receiving ICB. We found a trend that in patients treated with immunotherapy, the high PTTG1 expression group may have a longer survival time (although with the *p*-value >.05). Of importance, we found a significant difference in progression-free survival between low and high PTTG1 expression groups (Supplementary Figure 1B-C).

**Table 4.** Clinicopathological characteristics of pRCC patients treated with immunotherapy.

Variable	Entire group (*n* = 62)	*PTTG1* expression		*χ* ^2^	*p-*value
		Low expression (*n* = 19)	High expression (*n* = 43)		
Age at surgery (y, median ± SD)		58.5 ± 10.4	59.2 ± 12.9		
BMI (kg/m^2^, median ± SD)		22.5 ± 3.7	22.1 ± 3.1		
Tumour size (cm, median ± SD)		6.1 ± 0.8	5.9 ± 1.8		
OS time (month, median ± SD)		27.7 ± 8.3	50.0 ± 30.4		
PFS time (month, median ± SD)		25.0 ± 10.0	41.7 ± 29.8		
Sex (*n*, %)				0.904	.342
Male	39, 62.9%	12, 63.2%	27, 62.8%		
Female	23, 37.1%	7, 36.8%	16, 37.2%		
Laterality (*n*, %)				0.163	.687
Left	27, 43.5%	9, 47.4%	18, 41.9%		
Right	35, 56.5%	10, 52.6%	25, 58.1%		
Tumour type				0.011	.916
Type 1 pRCC	19, 30.6%	6, 31.6%	13, 30.2%		
Type 2 pRCC	43, 69.4%	13, 68.4%	30, 69.8%		
T stage at surgery (*n*, %)				1.361	.243
T1–T2	33, 53.2%	8, 42.1%	25, 58.1%		
T3–T4	29, 46.8%	11, 57.9%	18, 41.9%		
N stage at surgery (*n*, %)				0.001	.978
N0	39, 62.9%	12, 63.2%	27, 62.8%		
N1	23, 37.1%	7, 36.8%	16, 37.2%		
M stage at surgery (*n*, %)				0.072	.788
M0	38, 61.3%	12, 63.2%	25, 59.5%		
M1	24, 38.7%	7, 36.8%	17, 40.5%		
AJCC stage at surgery (*n*, %)				1.862	.172
I–II	17, 27.4%	3, 15.8%	14, 32.6%		
III–IV	45, 72.6%	16, 84.2%	29, 67.4%		
Furhman grade (*n*, %)				1.925	.165
1–2	3, 4.8%	2, 10.5%	1, 2.3%		
3–4	59, 95.2%	17, 89.5%	42, 97.7%		
ICB response (*n* = 62)				3.999	**.046**
PD/SD	41, 66.1%	16, 84.2%	25, 58.1%		
PR/CR	21, 33.9%	3, 15.8%	18, 41.9%		

pRCC: papillary renal cell carcinoma; BMI, body mass index; AJCC: American Joint Committee on Cancer; ICB: immune checkpoint blockade. *p-value less than .05 was considered statistically significant and marked in bold.

**Table 5.** Univariate Cox regression analyses of OS and PFS in 126 enrolled pRCC patients from FUSCC cohort.

	OS		PFS	
Covariates	HR (95%CI)	*p-*value	HR (95%CI)	*p-*value
Age at surgery	1.02 (1.00, 1.05)	.08	1.02 (1.00, 1.04)	.17
BMI (kg/m^2^)	0.83 (0.75, 0.92)	**<.01**	0.87 (0.79, 0.95)	**<.01**
Tumour size (cm)	1.17 (1.07, 1.29)	**<.01**	1.17 (1.08, 1.28)	**<.01**
Laterality (Ref. left)	0.75 (0.41, 1.35)	.33	0.97 (0.55, 1.73)	.92
T stage (T1-T2)	1.88 (1.02, 3.45)	**.04**	1.90 (1.05, 3.43)	**.03**
N stage (Ref. N0)	5.37 (2.91, 9.91)	**<.01**	5.75 (3.18, 10.40)	**<.01**
M stage (Ref. M0)	14.21 (7.24, 27.86)	**<.01**	14.36 (7.50, 27.47)	**<.01**
AJCC stage	7.52 (3.67, 15.43)	**<.01**	6.30 (3.25, 12.23)	**<.01**
Furhman grade (Ref. 1–2)	1.43 (0.64, 3.20)	.34	1.23 (0.59, 2.54)	.58
*PTTG1* expression (Ref. Low)	2.90 (1.52, 5.55)	**<.01**	2.46 (1.35, 4.51)	**<.01**

PFS: progression-free survival; pRCC: papillary renal cell carcinoma; FUSCC: Fudan University Shanghai Cancer Centre; HR: hazard ratio; CI: confidence interval; BMI: body mass index; *p-value less than .05 was considered as statistically significant and marked in bold.

**Table 6.** C-indexes of multivariate cox models in FUSCC cohort.

Variables in multivariate cox model	C-index	Standard deviation
T stage, N stage, M stage	0.6339	0.03411
T stage, N stage, M stage, expression level of PTTG1	0.6722	0.04166

## Discussion

The concept of pRCC was first proposed by Mancilla-Jimenez in 1976, thirty-four cases of RCC showed papillary structures. Of these, 85.3% pRCC patients have a better prognosis than other types of RCC [25]. Since pRCC is relatively rare in clinical practice and has been rarely studied, major molecular mechanisms in the pathogenesis are poorly understood. Therefore, potential biomarkers for efficient diagnosis and treatment are urgently needed. In this study, a total of 473 DEGs and 38 hub genes were identified by microarray data analysis. Among the 38 hub genes, 8 genes relating to cell cycle including *BUB1B*, *CCNB1*, *CCNB2*, *MAD2L1*, *TTK*, *CDC20*, *PTTG1*, *MCM5* were of prognostic value.

*BUB1B* (spindle detection point protein, also known as *BUBR1)* is an important functional protein at the detection point of mitosis and the change of *BUB1B* expression plays an important role in tumorigenesis and progression [26]. Studies have found that *BUB1B* is overexpressed in kidney cancer and breast cancer, its mutation and overexpression are strongly associated with Chromosomal instability [26,27]. A recent study [28] found that BUB1B overexpression is an independent prognostic marker in renal cell carcinoma, which is similar to our findings. Thus, it may be of great importance to further explore the biological significance of BUB1B in pRCC.

As a member of the cell cycle family, *CCNB1* is one of the key factors related to cell detection points [29,30]. Currently, Cyclin B1 overexpression has been found in a variety of human tumours, such as oesophageal cancer, non-small cell lung cancer, tongue cancer, and is related to tumour grade, differentiation, invasion, and metastasis, and prognosis [31]. Thus, there is enough evidence to doubt the role of *CCNB1* in human RCC as an oncogene.

It has been reported that *CCNB2* is highly expressed in tumour tissues, such as breast cancer [32], adrenal cortical carcinoma [33], colorectal adenocarcinoma [34], and pituitary adenoma [35]. It has also been reported that serum circulating *CCNB2* mRNA level in cancer patients is significantly higher than that in the normal population and benign diseases [36]. Thus, *CCNB2* may have played an important role in the generation and development of pRCC.

It has been shown that interrupting the function of *MAD2L1* in mammalian cells can affect the process of spindle examination and lead to the development of aneuploid cells or tumours. The deletion of the *MAD2L1* gene can cause chromosome instability and drive the development of tumours. In mouse models, deletion of the *MAD2L1* gene can cause liver and lung cancer [37].

*TTK* is the basic component of spindle assembly checkpoint(SAC), it plays an important role in the replication of mitotic centrosomes and the correct separation of chromosomes [38]. To maintain the division and proliferation of tumour cells, *TTK* was highly expressed in tumour cells to maintain the normal function of SAC. After inhibiting the function of *TTK*, SAC is damaged, errors in mitotic metaphase cannot be detected, chromosomes cannot be separated into daughter cells on average, and heteroploidy is further increased, exceeding a certain threshold will cause tumour cell apoptosis, so *TTK* can serve as an effective anti-tumour target [39,40].

Multiple studies have shown that *CDC20* could degrade several important substrate factors to regulate cell cycle progression including Securin [41], Cyclin A [42,43], p21 [44], and Mcl-1 [45]. The protein p21 is considered to be an effector of various tumour inhibition signalling pathways, partly deactivating Cyclin-dependent kinases to promote anti-tumour proliferation. Downregulation of p21 expression was detected in a variety of human malignancies, so *CDC20* may play its carcinogenic role in part by degrading tumour suppressor protein p21. Thus, the generation of pRCC may be caused partly by *CDC20*.

*MCM*s protein is closely related to the cell cycle. It is a promoter of DNA replication and plays a key role in regulating cells going from the G0 phase to the S phase [46]. As one of the *MCM* protein families, *MCM5* protein is closely related to cell proliferation. The expression of *MCM*s protein can be indirectly understood through the detection of *MCM5*. Williams et al. reported the application of *MCM5* protein antibody to diagnose abnormal prodromal malignant cells in pap cervical smear and found that the *MCM* had high sensitivity and specificity in detecting prodromal malignant cells in cervical smear [47]. Going has proposed this conclusion in oesophageal tissues [48]. These results indicate that *MCM5* protein has some predictive value.

*PTTG1* is a tumour transforming gene, which can cause cell transformation without the participation of any auxiliary gene, and is closely related to the occurrence of many tumours [49]. *PTTG1* has been identified as an oncogene. The expression level of *PTTG1* is closely related to tumour formation, angiogenesis, and metastasis [50]. In this research, we found that *PTTG1* exhibited strong associations with TME and we speculated that it may influence the anti-tumour immune due to various mechanisms. A higher expression level of *PTTG1* may indicate a higher infiltration of T cells. Since T cells play a key role in antitumor immunity, *PTTG1* may be a biomarker for predicting ICB response. Thus, we detected the expression level of PTTG1 protein in pRCC from the FUSCC cohort by using immunohistochemical staining and we found elevated PTTG1 expression in tumour tissues, and higher expression of *PTTG1* is significantly relevant to both OS and PFS. In addition, the retrospective analysis indicated that elevated PTTG1 expression was significantly associated with better ICB response, which implicated PTTG1 may serve as a potential biomarker for immunotherapy in pRCC.

But there are still several limitations in this study. First, the data utilized in the study consisted of unbalanced pRCC and control normal samples, which were restricted in quantity and downloaded from the Gene Expression Omnibus database. The chip data contains relatively small pRCC samples in a public database and only 323 patients were enrolled from the TCGA cohort with corresponding RNA sequence. Second, we only verified the prognostic and therapeutic significance of *PTTG1* in the FUSCC cohort, but the potential mechanism of the signalling pathway in pRCC is still not clear, while a series of functional annotations and enrichment analyses have been carried out. Thus, the detailed mechanism between the eight genes and pRCC needs to be further studied and that will be our next stage of works.

## Conclusion

In conclusion, the transcription profiles of *BUB1B*, *CCNB1*, *CCNB2*, *MAD2L1*, *TTK*, *CDC20*, *PTTG1*, and *MCM5* are prognostic and may contribute to a better understanding of the potential carcinogenesis and progression of pRCC. PTTG1 may also serve as a potential biomarker for immunotherapy in pRCC and further researches is also needed to elucidate the molecular mechanism and signalling pathway changes of these genes in pRCC.

## Supplementary Material

Supplemental Material

## Data Availability

The datasets during and/or analyzed during the current study are available from the corresponding author on reasonable request. Data from the TCGA cohort and GEO is public.

## Abbreviations

ccRCC: clear cell renal cell carcinoma
DEGs: differentially expressed genes
FUSCC: Fudan University Shanghai Cancer Centre
ICB: immune checkpoint blockade
pRCC: papillary renal cell carcinoma
PPI: protein-protein interaction network
RCC: renal cell carcinoma
