# Supplementary material for: Identification of prognostic biomarkers in papillary renal cell carcinoma and PTTG1 may serve as a biomarker for predicting immunotherapy response
Source: Ann Med. 2022 Jan 17;54(1):211–26. doi: 10.1080/07853890.2021.2011956 (PMC8765283; doi:10.1080/07853890.2021.2011956)
Supplement: Supplemental Material [file IANN_A_2011956_SM0583.zip › Supplemental files/TableS1.docx]

| NAME | GS<br> follow link to MSigDB | SIZE | ES | NES | NOM p-val | FDR q-val | FWER p-val | RANK AT MAX | LEADING EDGE |
| --- | --- | --- | --- | --- | --- | --- | --- | --- | --- |
| GO_VIRAL_GENE_EXPRESSION | GO_VIRAL_GENE_EXPRESSION | 190 | 0.691088 | 2.253734 | 0 | 0.0082 | 0.006 | 2096 | tags=53%, list=11%, signal=58% |
| GO_NUCLEAR_TRANSCRIBED_MRNA_CATABOLIC_PROCESS | GO_NUCLEAR_TRANSCRIBED_MRNA_CATABOLIC_PROCESS | 202 | 0.660532 | 2.235218 | 0 | 0.007615 | 0.009 | 2167 | tags=49%, list=11%, signal=54% |
| GO_NEGATIVE_REGULATION_OF_CHROMOSOME_SEGREGATION | GO_NEGATIVE_REGULATION_OF_CHROMOSOME_SEGREGATION | 44 | 0.746342 | 2.203455 | 0 | 0.012267 | 0.02 | 1335 | tags=45%, list=7%, signal=49% |
| GO_CYTOSOLIC_PART | GO_CYTOSOLIC_PART | 241 | 0.605496 | 2.171735 | 0 | 0.014693 | 0.033 | 2715 | tags=43%, list=14%, signal=49% |
| GO_SPINDLE_MIDZONE | GO_SPINDLE_MIDZONE | 34 | 0.701116 | 2.170134 | 0 | 0.012006 | 0.033 | 1067 | tags=35%, list=5%, signal=37% |
| GO_TRANSLATIONAL_INITIATION | GO_TRANSLATIONAL_INITIATION | 192 | 0.686876 | 2.162572 | 0 | 0.012561 | 0.041 | 1234 | tags=46%, list=6%, signal=48% |
| GO_KINETOCHORE_ORGANIZATION | GO_KINETOCHORE_ORGANIZATION | 21 | 0.845242 | 2.162369 | 0 | 0.010767 | 0.041 | 1314 | tags=57%, list=7%, signal=61% |
| GO_PROTEIN_TARGETING_TO_MEMBRANE | GO_PROTEIN_TARGETING_TO_MEMBRANE | 192 | 0.635972 | 2.158109 | 0.002028 | 0.010516 | 0.044 | 1864 | tags=45%, list=9%, signal=49% |
| GO_NEGATIVE_REGULATION_OF_METAPHASE_ANAPHASE_TRANSITION_OF_CELL_CYCLE | GO_NEGATIVE_REGULATION_OF_METAPHASE_ANAPHASE_TRANSITION_OF_CELL_CYCLE | 37 | 0.74928 | 2.154367 | 0 | 0.010351 | 0.047 | 1335 | tags=46%, list=7%, signal=49% |
| GO_MITOTIC_SISTER_CHROMATID_SEGREGATION | GO_MITOTIC_SISTER_CHROMATID_SEGREGATION | 148 | 0.65817 | 2.15126 | 0 | 0.009316 | 0.047 | 1545 | tags=38%, list=8%, signal=41% |
| GO_REGULATION_OF_CHROMOSOME_SEPARATION | GO_REGULATION_OF_CHROMOSOME_SEPARATION | 61 | 0.695645 | 2.143435 | 0 | 0.010736 | 0.056 | 1545 | tags=39%, list=8%, signal=43% |
| GO_MITOTIC_NUCLEAR_DIVISION | GO_MITOTIC_NUCLEAR_DIVISION | 279 | 0.579107 | 2.141246 | 0 | 0.010081 | 0.058 | 1612 | tags=31%, list=8%, signal=33% |
| GO_PROTEIN_LOCALIZATION_TO_ENDOPLASMIC_RETICULUM | GO_PROTEIN_LOCALIZATION_TO_ENDOPLASMIC_RETICULUM | 135 | 0.762982 | 2.135157 | 0.002016 | 0.010091 | 0.064 | 2301 | tags=64%, list=12%, signal=72% |
| GO_RNA_CATABOLIC_PROCESS | GO_RNA_CATABOLIC_PROCESS | 391 | 0.551438 | 2.134237 | 0 | 0.009499 | 0.065 | 2314 | tags=37%, list=12%, signal=41% |
| GO_NUCLEAR_TRANSCRIBED_MRNA_CATABOLIC_PROCESS_NONSENSE_MEDIATED_DECAY | GO_NUCLEAR_TRANSCRIBED_MRNA_CATABOLIC_PROCESS_NONSENSE_MEDIATED_DECAY | 119 | 0.783435 | 2.127427 | 0 | 0.010386 | 0.073 | 1153 | tags=63%, list=6%, signal=67% |
| GO_CONDENSED_CHROMOSOME | GO_CONDENSED_CHROMOSOME | 218 | 0.60356 | 2.123838 | 0 | 0.010467 | 0.079 | 3175 | tags=42%, list=16%, signal=49% |
| GO_KINETOCHORE_ASSEMBLY | GO_KINETOCHORE_ASSEMBLY | 16 | 0.833435 | 2.122725 | 0 | 0.010239 | 0.08 | 1160 | tags=50%, list=6%, signal=53% |
| GO_CONDENSED_CHROMOSOME_CENTROMERIC_REGION | GO_CONDENSED_CHROMOSOME_CENTROMERIC_REGION | 114 | 0.687218 | 2.119698 | 0 | 0.010122 | 0.084 | 1366 | tags=40%, list=7%, signal=43% |
| GO_CENTROMERE_COMPLEX_ASSEMBLY | GO_CENTROMERE_COMPLEX_ASSEMBLY | 54 | 0.673225 | 2.119551 | 0 | 0.009656 | 0.085 | 1358 | tags=37%, list=7%, signal=40% |
| GO_METAPHASE_ANAPHASE_TRANSITION_OF_CELL_CYCLE | GO_METAPHASE_ANAPHASE_TRANSITION_OF_CELL_CYCLE | 56 | 0.704951 | 2.118455 | 0 | 0.009356 | 0.087 | 1545 | tags=41%, list=8%, signal=44% |
| GO_SPINDLE_MICROTUBULE | GO_SPINDLE_MICROTUBULE | 54 | 0.678316 | 2.115017 | 0 | 0.009359 | 0.089 | 1609 | tags=37%, list=8%, signal=40% |
| GO_REGULATION_OF_NUCLEAR_DIVISION | GO_REGULATION_OF_NUCLEAR_DIVISION | 206 | 0.557692 | 2.112723 | 0 | 0.009765 | 0.099 | 3867 | tags=42%, list=20%, signal=52% |
| GO_KINETOCHORE | GO_KINETOCHORE | 130 | 0.66209 | 2.09638 | 0 | 0.012935 | 0.12 | 1366 | tags=38%, list=7%, signal=40% |
| GO_CHROMATIN_REMODELING_AT_CENTROMERE | GO_CHROMATIN_REMODELING_AT_CENTROMERE | 46 | 0.677312 | 2.087613 | 0 | 0.013958 | 0.129 | 3019 | tags=50%, list=15%, signal=59% |
| GO_POSITIVE_REGULATION_OF_NUCLEAR_DIVISION | GO_POSITIVE_REGULATION_OF_NUCLEAR_DIVISION | 66 | 0.555194 | 2.086398 | 0 | 0.013745 | 0.132 | 2661 | tags=33%, list=14%, signal=38% |
| GO_SISTER_CHROMATID_SEGREGATION | GO_SISTER_CHROMATID_SEGREGATION | 180 | 0.627862 | 2.085086 | 0 | 0.013425 | 0.133 | 1545 | tags=35%, list=8%, signal=38% |
| GO_NEGATIVE_REGULATION_OF_CELL_CYCLE_PHASE_TRANSITION | GO_NEGATIVE_REGULATION_OF_CELL_CYCLE_PHASE_TRANSITION | 234 | 0.530057 | 2.081034 | 0 | 0.014161 | 0.141 | 3764 | tags=44%, list=19%, signal=54% |
| GO_SMN_SM_PROTEIN_COMPLEX | GO_SMN_SM_PROTEIN_COMPLEX | 17 | 0.838325 | 2.079811 | 0 | 0.013984 | 0.144 | 1689 | tags=65%, list=9%, signal=71% |
| GO_NEGATIVE_REGULATION_OF_NUCLEAR_DIVISION | GO_NEGATIVE_REGULATION_OF_NUCLEAR_DIVISION | 59 | 0.649936 | 2.079332 | 0 | 0.0136 | 0.144 | 1464 | tags=39%, list=7%, signal=42% |
| GO_NUCLEAR_CHROMOSOME_SEGREGATION | GO_NUCLEAR_CHROMOSOME_SEGREGATION | 252 | 0.579354 | 2.071011 | 0 | 0.01544 | 0.154 | 2043 | tags=33%, list=10%, signal=36% |
| GO_ANAPHASE_PROMOTING_COMPLEX_DEPENDENT_CATABOLIC_PROCESS | GO_ANAPHASE_PROMOTING_COMPLEX_DEPENDENT_CATABOLIC_PROCESS | 81 | 0.661827 | 2.066379 | 0 | 0.016172 | 0.159 | 2312 | tags=47%, list=12%, signal=53% |
| GO_CONDENSED_NUCLEAR_CHROMOSOME | GO_CONDENSED_NUCLEAR_CHROMOSOME | 99 | 0.579495 | 2.052542 | 0 | 0.020268 | 0.201 | 3175 | tags=38%, list=16%, signal=46% |
| GO_CHROMOSOME_CENTROMERIC_REGION | GO_CHROMOSOME_CENTROMERIC_REGION | 190 | 0.620743 | 2.048893 | 0 | 0.020721 | 0.211 | 1401 | tags=34%, list=7%, signal=36% |
| GO_HISTONE_EXCHANGE | GO_HISTONE_EXCHANGE | 58 | 0.621828 | 2.045166 | 0 | 0.021021 | 0.218 | 3019 | tags=43%, list=15%, signal=51% |
| GO_CONDENSED_NUCLEAR_CHROMOSOME_CENTROMERIC_REGION | GO_CONDENSED_NUCLEAR_CHROMOSOME_CENTROMERIC_REGION | 24 | 0.764119 | 2.044842 | 0 | 0.020477 | 0.22 | 1182 | tags=50%, list=6%, signal=53% |
| GO_CHROMOSOME_SEGREGATION | GO_CHROMOSOME_SEGREGATION | 304 | 0.567958 | 2.041432 | 0 | 0.020886 | 0.23 | 1562 | tags=30%, list=8%, signal=32% |
| GO_ESTABLISHMENT_OF_PROTEIN_LOCALIZATION_TO_MEMBRANE | GO_ESTABLISHMENT_OF_PROTEIN_LOCALIZATION_TO_MEMBRANE | 321 | 0.509114 | 2.041312 | 0.002058 | 0.020321 | 0.23 | 2418 | tags=35%, list=12%, signal=39% |
| GO_POSITIVE_REGULATION_OF_MITOTIC_NUCLEAR_DIVISION | GO_POSITIVE_REGULATION_OF_MITOTIC_NUCLEAR_DIVISION | 54 | 0.575567 | 2.041227 | 0 | 0.019786 | 0.23 | 2580 | tags=35%, list=13%, signal=40% |
| GO_CHROMOSOME_SEPARATION | GO_CHROMOSOME_SEPARATION | 88 | 0.62222 | 2.040387 | 0 | 0.01963 | 0.231 | 1545 | tags=33%, list=8%, signal=36% |
| GO_REGULATION_OF_SISTER_CHROMATID_SEGREGATION | GO_REGULATION_OF_SISTER_CHROMATID_SEGREGATION | 79 | 0.645619 | 2.039948 | 0 | 0.019421 | 0.234 | 1545 | tags=37%, list=8%, signal=40% |
| GO_MITOTIC_METAPHASE_PLATE_CONGRESSION | GO_MITOTIC_METAPHASE_PLATE_CONGRESSION | 44 | 0.67458 | 2.031781 | 0 | 0.021449 | 0.256 | 1235 | tags=39%, list=6%, signal=41% |
| GO_ESTABLISHMENT_OF_PROTEIN_LOCALIZATION_TO_ENDOPLASMIC_RETICULUM | GO_ESTABLISHMENT_OF_PROTEIN_LOCALIZATION_TO_ENDOPLASMIC_RETICULUM | 111 | 0.812733 | 2.028144 | 0.002012 | 0.022257 | 0.268 | 2301 | tags=76%, list=12%, signal=85% |
| GO_NEGATIVE_REGULATION_OF_CELL_CYCLE_PROCESS | GO_NEGATIVE_REGULATION_OF_CELL_CYCLE_PROCESS | 325 | 0.499596 | 2.024483 | 0 | 0.022897 | 0.273 | 3806 | tags=41%, list=19%, signal=50% |
| GO_DNA_REPLICATION_INDEPENDENT_NUCLEOSOME_ORGANIZATION | GO_DNA_REPLICATION_INDEPENDENT_NUCLEOSOME_ORGANIZATION | 55 | 0.637966 | 2.022994 | 0.002012 | 0.022592 | 0.274 | 3019 | tags=45%, list=15%, signal=54% |
| GO_SM_LIKE_PROTEIN_FAMILY_COMPLEX | GO_SM_LIKE_PROTEIN_FAMILY_COMPLEX | 75 | 0.634848 | 2.022556 | 0 | 0.022326 | 0.275 | 1960 | tags=39%, list=10%, signal=43% |
| GO_POSITIVE_REGULATION_OF_CELL_CYCLE_PHASE_TRANSITION | GO_POSITIVE_REGULATION_OF_CELL_CYCLE_PHASE_TRANSITION | 93 | 0.562413 | 2.017464 | 0 | 0.023499 | 0.289 | 3745 | tags=41%, list=19%, signal=50% |
| GO_NEGATIVE_REGULATION_OF_CELL_CYCLE_G2_M_PHASE_TRANSITION | GO_NEGATIVE_REGULATION_OF_CELL_CYCLE_G2_M_PHASE_TRANSITION | 101 | 0.581857 | 2.009514 | 0 | 0.025671 | 0.308 | 3764 | tags=52%, list=19%, signal=65% |
| GO_ORGANELLE_FISSION | GO_ORGANELLE_FISSION | 454 | 0.493753 | 2.002291 | 0 | 0.027826 | 0.333 | 2074 | tags=27%, list=11%, signal=30% |
| GO_RESPONSE_TO_INTERLEUKIN_12 | GO_RESPONSE_TO_INTERLEUKIN_12 | 50 | 0.585549 | 1.999986 | 0 | 0.028324 | 0.343 | 2722 | tags=44%, list=14%, signal=51% |
| GO_ROUGH_ENDOPLASMIC_RETICULUM_MEMBRANE | GO_ROUGH_ENDOPLASMIC_RETICULUM_MEMBRANE | 27 | 0.636333 | 1.998326 | 0 | 0.028224 | 0.349 | 2925 | tags=44%, list=15%, signal=52% |
| GO_U1_SNRNP | GO_U1_SNRNP | 18 | 0.821802 | 1.998293 | 0 | 0.027771 | 0.35 | 574 | tags=50%, list=3%, signal=51% |
| GO_REGULATION_OF_CHROMOSOME_SEGREGATION | GO_REGULATION_OF_CHROMOSOME_SEGREGATION | 100 | 0.625966 | 1.995059 | 0 | 0.028511 | 0.363 | 1545 | tags=36%, list=8%, signal=39% |
| GO_METAPHASE_PLATE_CONGRESSION | GO_METAPHASE_PLATE_CONGRESSION | 57 | 0.62812 | 1.994759 | 0 | 0.02817 | 0.365 | 1235 | tags=33%, list=6%, signal=35% |
| GO_REGULATION_OF_UBIQUITIN_PROTEIN_LIGASE_ACTIVITY | GO_REGULATION_OF_UBIQUITIN_PROTEIN_LIGASE_ACTIVITY | 21 | 0.705307 | 1.991217 | 0 | 0.029425 | 0.374 | 2074 | tags=57%, list=11%, signal=64% |
| GO_REGULATION_OF_CYCLIN_DEPENDENT_PROTEIN_KINASE_ACTIVITY | GO_REGULATION_OF_CYCLIN_DEPENDENT_PROTEIN_KINASE_ACTIVITY | 100 | 0.529935 | 1.990154 | 0 | 0.029224 | 0.375 | 3519 | tags=41%, list=18%, signal=50% |
| GO_ATTACHMENT_OF_SPINDLE_MICROTUBULES_TO_KINETOCHORE | GO_ATTACHMENT_OF_SPINDLE_MICROTUBULES_TO_KINETOCHORE | 32 | 0.726025 | 1.989816 | 0 | 0.028855 | 0.376 | 1235 | tags=50%, list=6%, signal=53% |
| GO_CYTOSOLIC_SMALL_RIBOSOMAL_SUBUNIT | GO_CYTOSOLIC_SMALL_RIBOSOMAL_SUBUNIT | 44 | 0.849776 | 1.989521 | 0 | 0.028563 | 0.378 | 782 | tags=73%, list=4%, signal=76% |
| GO_DNA_REPLICATION_INITIATION | GO_DNA_REPLICATION_INITIATION | 37 | 0.743645 | 1.982762 | 0 | 0.030792 | 0.403 | 1674 | tags=54%, list=9%, signal=59% |
| GO_SPLICEOSOMAL_SNRNP_ASSEMBLY | GO_SPLICEOSOMAL_SNRNP_ASSEMBLY | 38 | 0.684053 | 1.9821 | 0 | 0.030499 | 0.406 | 1960 | tags=50%, list=10%, signal=55% |
| GO_PROTEASOMAL_UBIQUITIN_INDEPENDENT_PROTEIN_CATABOLIC_PROCESS | GO_PROTEASOMAL_UBIQUITIN_INDEPENDENT_PROTEIN_CATABOLIC_PROCESS | 23 | 0.769875 | 1.972155 | 0.00202 | 0.0348 | 0.435 | 2927 | tags=70%, list=15%, signal=82% |
| GO_MEIOTIC_CELL_CYCLE_PROCESS | GO_MEIOTIC_CELL_CYCLE_PROCESS | 184 | 0.530862 | 1.97127 | 0 | 0.034948 | 0.441 | 2661 | tags=32%, list=14%, signal=37% |
| GO_POLYSOMAL_RIBOSOME | GO_POLYSOMAL_RIBOSOME | 29 | 0.835554 | 1.959229 | 0 | 0.040757 | 0.489 | 782 | tags=66%, list=4%, signal=68% |
| GO_PROTEIN_LOCALIZATION_TO_CHROMOSOME_CENTROMERIC_REGION | GO_PROTEIN_LOCALIZATION_TO_CHROMOSOME_CENTROMERIC_REGION | 22 | 0.820526 | 1.957765 | 0 | 0.040938 | 0.492 | 1335 | tags=68%, list=7%, signal=73% |
| GO_COTRANSLATIONAL_PROTEIN_TARGETING_TO_MEMBRANE | GO_COTRANSLATIONAL_PROTEIN_TARGETING_TO_MEMBRANE | 98 | 0.835678 | 1.957528 | 0.004008 | 0.040408 | 0.494 | 2301 | tags=84%, list=12%, signal=94% |
| GO_NEGATIVE_REGULATION_OF_MITOTIC_CELL_CYCLE | GO_NEGATIVE_REGULATION_OF_MITOTIC_CELL_CYCLE | 305 | 0.479953 | 1.955162 | 0 | 0.041099 | 0.498 | 2186 | tags=30%, list=11%, signal=34% |
| GO_CYCLIN_DEPENDENT_PROTEIN_SERINE_THREONINE_KINASE_REGULATOR_ACTIVITY | GO_CYCLIN_DEPENDENT_PROTEIN_SERINE_THREONINE_KINASE_REGULATOR_ACTIVITY | 47 | 0.581681 | 1.948708 | 0 | 0.043598 | 0.516 | 3519 | tags=47%, list=18%, signal=57% |
| GO_REGULATION_OF_CELL_CYCLE_PHASE_TRANSITION | GO_REGULATION_OF_CELL_CYCLE_PHASE_TRANSITION | 434 | 0.477197 | 1.946105 | 0 | 0.044606 | 0.529 | 3764 | tags=36%, list=19%, signal=43% |
| GO_REGULATION_OF_CELL_CYCLE_G2_M_PHASE_TRANSITION | GO_REGULATION_OF_CELL_CYCLE_G2_M_PHASE_TRANSITION | 211 | 0.51791 | 1.945909 | 0.002041 | 0.044084 | 0.531 | 3764 | tags=40%, list=19%, signal=49% |
| GO_PRONUCLEUS | GO_PRONUCLEUS | 16 | 0.764686 | 1.943355 | 0.001984 | 0.045001 | 0.544 | 1567 | tags=50%, list=8%, signal=54% |
| GO_MITOTIC_CELL_CYCLE_CHECKPOINT | GO_MITOTIC_CELL_CYCLE_CHECKPOINT | 163 | 0.543309 | 1.939961 | 0 | 0.04606 | 0.554 | 1662 | tags=32%, list=8%, signal=35% |
| GO_U2_SNRNP | GO_U2_SNRNP | 21 | 0.743175 | 1.936703 | 0.002033 | 0.047282 | 0.563 | 574 | tags=48%, list=3%, signal=49% |
| GO_CELL_CYCLE_G2_M_PHASE_TRANSITION | GO_CELL_CYCLE_G2_M_PHASE_TRANSITION | 265 | 0.508926 | 1.93509 | 0.00207 | 0.04762 | 0.567 | 3764 | tags=39%, list=19%, signal=48% |
| GO_CYCLIN_DEPENDENT_PROTEIN_KINASE_HOLOENZYME_COMPLEX | GO_CYCLIN_DEPENDENT_PROTEIN_KINASE_HOLOENZYME_COMPLEX | 41 | 0.637721 | 1.934737 | 0 | 0.047143 | 0.568 | 3494 | tags=51%, list=18%, signal=62% |
| GO_PROTEIN_LOCALIZATION_TO_KINETOCHORE | GO_PROTEIN_LOCALIZATION_TO_KINETOCHORE | 17 | 0.831647 | 1.933772 | 0 | 0.047171 | 0.574 | 1944 | tags=76%, list=10%, signal=85% |
| GO_THREONINE_TYPE_PEPTIDASE_ACTIVITY | GO_THREONINE_TYPE_PEPTIDASE_ACTIVITY | 21 | 0.792131 | 1.930249 | 0.004124 | 0.048778 | 0.583 | 2927 | tags=76%, list=15%, signal=89% |
| GO_U12_TYPE_SPLICEOSOMAL_COMPLEX | GO_U12_TYPE_SPLICEOSOMAL_COMPLEX | 26 | 0.70611 | 1.929615 | 0 | 0.048539 | 0.584 | 1689 | tags=50%, list=9%, signal=55% |
| GO_NUCLEAR_UBIQUITIN_LIGASE_COMPLEX | GO_NUCLEAR_UBIQUITIN_LIGASE_COMPLEX | 43 | 0.582638 | 1.928456 | 0.002053 | 0.048789 | 0.588 | 3569 | tags=44%, list=18%, signal=54% |
| GO_DNA_PACKAGING | GO_DNA_PACKAGING | 202 | 0.518726 | 1.927131 | 0 | 0.049135 | 0.593 | 4746 | tags=45%, list=24%, signal=58% |
